# Liver Mitochondrial Transcriptomic Responses to Dietary Crude Protein and Phosphorus Deficiencies and Feed Restriction in Wethers

**DOI:** 10.3390/genes17060644

**Published:** 2026-05-31

**Authors:** Elmer E. Fernandez, David J. Innes, Walter G. Bottje, Marina R. S. Fortes, Dennis P. Poppi, Simon P. Quigley, Jude J. Bond, Nicholas J. Hudson

**Affiliations:** 1 School of Agriculture and Food Sustainability, The University of Queensland, Gatton, QLD 4343, Australia; e.fernandezguerra@uq.edu.au (E.E.F.); d.poppi@uq.edu.au (D.P.P.); 2 Department of Animal Sciences, University of New England, Armidale, NSW 2350, Australia; david.innes@une.edu.au; 3 Department of Poultry Science, University of Arkansas, Fayetteville, AR 72701, USA; wbottje@uark.edu; 4 School of Chemistry and Molecular Biosciences, The University of Queensland, Brisbane, QLD 4072, Australia; m.fortes@uq.edu.au; 5 Institute for Future Farming Systems, Central Queensland University, Rockhampton, QLD 4701, Australia; s.quigley@cqu.edu.au; 6 Extensive Livestock Industry Centre, New South Wales Department of Primary Industries and Rural Development, Armidale, NSW 2351, Australia; judevandeurzen@gmail.com

**Keywords:** folate-mediated one-carbon metabolism, nitrogen-sparing amino-acid catabolism, mitochondrial solute carriers, phosphocreatine energy buffering, ruminant nutrition

## Abstract

**Background/Objectives:** Seasonal crude protein (CP) and phosphorus (P) deficiency in northern Australian pastures reduces feed intake and growth of grazing ruminants, but the hepatic mitochondrial mechanisms underlying this response remain unclear. We characterized the hepatic mitochondrial transcriptome of sheep exposed to CP-P deficiency or matched-intake feed restriction. **Methods:** Merino wethers were assigned for 63 d to one of three treatments (*n* = 8/group): High CP-P, Low CP-P, or Restricted, in which High CP-P feed was offered at the same energy intake as the Low CP-P group. Liver RNA was sequenced, and transcripts encoding mitochondrial proteins were identified using MitoCarta 3.0. Differentially expressed genes (DEGs) were defined as adjusted *p* < 0.05 and |log2FC| ≥ 0.585. **Results:** Of 804 mitochondrial genes detected, 83 were differentially expressed in at least one pairwise comparison. The greatest transcriptional response occurred in contrasts against High CP-P (Low CP-P vs. High CP-P: 38 DEGs in 8 enriched pathways; Restricted vs. High CP-P: 37 DEGs in 10 enriched pathways). In both low-intake treatments, *ALDH1L2*, *ALDH1L1*, *SHMT2*, and *DMGDH* were upregulated, suggesting altered folate-mediated one-carbon metabolism. Restricted sheep also showed higher expression of several *SLC25A* transporters (*SLC25A4*, *SLC25A28*, *SLC25A29*, *SLC25A33*, and *SLC25A34*), indicative of enhanced mitochondrial nucleotide and metabolite exchange under CP-P adequate energy restriction. In contrast, Low CP-P sheep showed higher expression of *SLC25A15* and *SLC25A25* relative to either High CP-P or Restricted sheep, a nutrient-deficiency specific transporter response. *CKMT2* expression was also higher in Restricted sheep than in both other groups. **Conclusions:** These findings suggest that reduced metabolizable energy intake was associated with the bulk of the hepatic mitochondrial transcriptional response, particularly in folate-mediated one-carbon metabolism, whereas CP-P deficiency was associated with a smaller but distinct transporter signature. The liver mitochondrial transcriptome may provide mechanistic insight into nutritional adaptation under CP and P deficiency in grazing sheep.

## 1. Introduction

Extensive grazing systems across the world, including regions of Australia, Brazil, South Africa, and the southern US, pose significant nutritional challenges for sheep due to pronounced seasonal fluctuations in forage quality. During the dry season, forages mature and pasture crude protein (CP) content often falls below maintenance requirements, commonly <7% CP [1], and soils can be characteristically low in phosphorus (P). Sheep grazing these pastures may exhibit reduced voluntary feed intake and body weight gain, and this loss of appetite has been attributed to metabolic feedback rather than a mechanical limitation in rumen fill [2,3]. Crude protein and P are both fundamental to normal animal growth and development. Phosphorus is required for skeletal development and has central roles in cellular metabolism, genetic storage and transfer, and membrane structure [4]. Crude protein supplies the protein and non-protein nitrogen to support rumen microbial growth, which is the primary source of amino acids for ruminant whole-body metabolism. When CP or P supply is inadequate, voluntary feed intake, microbial protein synthesis, and cellulose digestion can decline [5,6]. These deficiencies can also impair reproductive performance, therefore having broad negative consequences for livestock productivity and commercial outcomes [7,8].

Mitochondria are central to these adaptations because they integrate ATP production with fatty acid oxidation, amino-acid catabolism, and tricarboxylic acid cycle activity. Mitochondrial function is responsive to substrate supply and energy demand, and nutrient restriction in other species has been associated with altered mitochondrial respiration or content [9,10,11,12]. Despite this biological relevance, there has been limited direct investigation of how chronic CP or P deficiency may alter mitochondrial biology in ruminants.

Earlier work in ruminants has described phenotypic effects of nutritional deficiencies, and recent molecular studies have been applied to understand the mechanisms involved. Transcriptomic analysis of bovine liver showed that a low-protein, low-mineral diet altered pathways related to lipid metabolism, glutathione metabolism, and energy reserve mobilization [13]. In dairy cattle, transcriptomic and proteomic studies associated feed efficiency with differential expression of mitochondrial genes, including oxidative phosphorylation components that suggest more economical energy utilization [14]. However, studies directly examining mitochondrial content and function under defined nutrient restrictions have been sparse. To address this gap, authors of this study previously implemented a model of nutritional stress in Merino sheep to explore how liver mitochondria and other tissues respond to chronic CP and P deficiencies [2,15]. By measuring mitochondrial DNA (mtDNA) copy number as a proxy of mitochondrial abundance, the liver was identified as an organ whose mitochondrial population responds strongly to these nutritional challenges.

Transcriptomic analysis is a powerful tool to investigate mitochondrial adaptation to nutritional stress. Because over 1000 genes encode mitochondrial proteins (the majority are nuclear-encoded), changes in nutrient supply can elicit coordinated shifts in transcription of metabolic enzymes, transporters, and regulatory factors that reside in or affect mitochondrial metabolism [10]. These studies allow identification of key pathways and genes that could serve as indicators of metabolic stress or targets for nutritional intervention. A hypothalamic transcriptomic study in sheep fed a low CP/P diet found neuroendocrine responses similar to that of feed restricted animals (hunger), despite voluntary underfeeding, while also downregulating genes involved with oxidative phosphorylation, glycolysis, and the citric acid cycle [2]. In the context of mitochondria, transcriptomic data can reveal evidence of altered mitochondrial biogenesis, shifts in oxidative capacity, and remodeling of substrate metabolism [16,17]. Such changes reflect the animal’s attempt to maintain energy homeostasis under nutrient constraints. Moreover, concurrent measurement of mtDNA copy number (or mitochondrial protein markers) may provide a physical correlation to transcriptional changes, distinguishing whether an observed gene expression shift likely corresponds to a change in actual mitochondrial content or functional state.

The aim of this study is to examine how mitochondrial and mitochondria-associated gene expression are altered by CP and P deficiencies, representative of possible nutritional conditions of ruminants grazing dry season pastures low in CP and P, in comparison to both animals consuming an adequate or a feed-restricted diet.

## 2. Materials and Methods

### 2.1. Animal Management

All research protocols for this study were approved by The University of Queensland’s Animal Ethics Committee in accordance with the Australian Code of Practice for the Care and Use of Animals for Scientific Purposes, identified under AEC approval number SAFS/049/19 on 28 March 2019. Detailed methods for the feeding trial and tissue sampling have been previously described [2]). Briefly, forty Merino wethers (castrated male sheep) were sourced from and reared under identical conditions by a commercial supplier (Armidale, NSW, Australia) at approximately seven months of age, with an initial live weight of 21.4 ± 1.1 kg. Over 24 days, the sheep were acclimated and transitioned to a pelleted diet. Wethers were individually penned and fed one of five dietary treatments for 63 days, each designed to simulate pasture conditions typical of northern Australia. Four pelleted diets were fed (n = 8/group) ad libitum and varied in CP (high: 110 g/kg DM, low: 55 g/kg DM) and P (high: 2.5 g/kg DM, low: 0.7 g/kg DM) concentrations, all with a metabolizable energy (ME) content of 9 MJ/kg DM. The dietary treatments included High CP-P (sufficient CP and P), High CP-Low P, Low CP-High P, and Low CP-P. An additional restricted treatment (Restricted) involved restricting the intake of the High CP-P diet to match the ME intake observed in sheep fed the Low CP-P diet, in order to provide two groups of equal ME intake where one is experiencing voluntary reduction in feed intake due to nutrient deficiency, while the other is feed restricted. Ingredients and chemical compositions of these diets can be found in Appendix A Table A1.

### 2.2. Euthanasia and Tissue Collection

The wethers were euthanized over four consecutive days. All animals were fed their respective dietary treatment exactly 2 h before euthanasia. Wethers were euthanized by pentobarbitone overdose given via the jugular vein, and subsequent exsanguination. At harvest, liver samples were immediately collected, placed into cryogenic tubes, flash-frozen in liquid nitrogen, and stored at −80 °C until analysis.

### 2.3. Rna Extraction and Sequencing

Total RNA was extracted from approximately 100 mg of liver tissue using a combined TRIzol^®^ (Life Technologies; Carlsbad, CA, USA) and RNeasy Plus Mini Kit (Qiagen, Hilden, Germany) protocol, as previously detailed [2]. RNA integrity was assessed using an Agilent 2200 TapeStation, yielding RNA integrity numbers (RIN) of 9.1 ± 0.2. Complementary DNA libraries were prepared at the Ramaciotti Centre for Genomics (University of NSW, Sydney, NSW, Australia) using Illumina’s TruSeq Stranded mRNA Sample Preparation Kit according to the manufacturer’s protocol. Sequencing was performed on an Illumina NovaSeq 6000 platform, generating 100 base pair single-end reads with a target sequencing depth of at least 20 million reads per sample.

### 2.4. Data Processing, Alignment, and Counting

Raw FASTQ files underwent quality control using FASTQC (version 0.11.8) and were trimmed with Trimmomatic (version 0.36) to remove low-quality bases (Sliding Window: 4 bases, minimum quality Q15; minimum length 36 bases; trim leading and trailing three bases). Cleaned reads were mapped to the ovine reference genome Oar_v3.1 (Ensembl release 100) using STAR (version 2.6.0b). Gene-level counts were quantified using featureCounts aligned with the Ensembl annotation file (Oar_v3.1.100.gtf). The entire preprocessing pipeline was executed on the Australian Galaxy web platform. For liver samples, sequencing and alignment yielded an average of 86% of the ~22.3 million reads per sample were aligned to the ovine reference genome, and approximately 49% of these reads were assigned to a single gene, resulting in quantification of 22,882 out of 27,054 genes (85%) with more than 10 reads across all samples and were retained for downstream analysis.

### 2.5. Statistical Analysis and RNA-Seq Data Visualization

Pairwise comparisons of the raw count data were initially performed among the five dietary treatments (High CP-P; High CP-Low P; Low CP-High P; Low CP-P; and Restricted) using the DESeq2 package (version 1.28.1) in R (version 4.2.2; R Core Team, 2022) within RStudio (version 2022.12.0+353). For the present study, subsequent analyses were restricted to 24 sheep from three dietary treatments (High CP-P; Low CP-P; and Restricted), selected to provide the clearest contrasts for the study objectives: a nutritionally adequate diet (High CP-P), a combined CP and P deficiency (Low CP-P), and a restricted regimen of the adequate diet (Restricted). Genes were considered significantly differentially expressed (DEGs) if they showed a Benjamini–Hochberg adjusted *p*-value (padj) ≤ 0.05 and an absolute log2 fold-change (log_2_FC) ≥ 0.585 (representing a ≥ 1.5-fold difference in expression).

Volcano plots were generated to visually represent DEGs, highlighting fold-change thresholds and statistical significance using the ggplot2 and ggrepel packages. Mean-Average (MA) plots were similarly created to illustrate the relationship between average gene expression levels and their corresponding log_2_FC across dietary comparisons. The top 30 mitochondria-associated DEGs by significance (lowest padj) in each dietary contrast were specifically annotated for clarity.

Hierarchical clustering and heatmaps of significantly expressed mitochondrial-associated genes across the 24 individual sheep were constructed using variance-stabilized log_2_-normalized gene expression values obtained via the DESeq2 variance stabilization transformation (VST). For gene-wise hierarchical clustering, expression values were converted to Z-scores and clustering was performed using Euclidean distance and complete linkage methods implemented in the pheatmap package (version 1.0.12). The resulting dendrograms and heatmaps visualized sample groupings and dietary effects on mitochondrial gene expression profiles.

### 2.6. Mitochondrial Gene Annotation and Pathway Enrichment Analysis

This study focused on DEGs identified within the MitoCarta 3.0 database [18], which categorizes 1036 mitochondrial associated genes into manually curated functional pathways.

Functional enrichment analyses were performed using a hypergeometric test implemented through custom R scripts. The hypergeometric test assessed the over-representation of mitochondrial pathways within the DEGs from each dietary comparison relative to the background mitochondrial gene set. Significance thresholds for pathway enrichment were set at Benjamini–Hochberg adjusted *p*-values < 0.05.

A dot-plot visualization was produced to summarize mitochondrial pathway enrichment across dietary comparisons. Dot sizes represented the number of significant DEGs per pathway, while dot colors indicated statistical significance (−log_10_ adjusted *p*-values). Additionally, a pathway-annotated heatmap was generated for DEGs within enriched mitochondrial pathways, clearly visualizing expression changes across dietary contrasts. Rows (genes) were annotated with the most specific mitochondrial pathways identified.

### 2.7. Mitochondrial Co-Expression Network Mapping and Transcriptome Overlay

Transcriptome data were mapped across a high-resolution bovine mitochondrial co-expression network [19]. This network was reverse-engineered from 723 RNA-seq datasets spanning 91 tissues and cell types in cattle, and comprises over 870 mRNA nodes and >12,000 edges, representing highly conserved, partially correlated gene–gene interactions within Cytoscape (v3.10.0) [20] across the mitochondrial proteome.

For visualization and biological interpretation, gene symbols for all genes identified in the present study were mapped onto the corresponding nodes within the network. Expression log2 fold changes (relative to dietary treatment contrast) were overlaid onto the network using Cytoscape’s continuous node color mapping function, enabling the identification of pathway- or module-level regulatory responses.

## 3. Results

**Figure 1:**
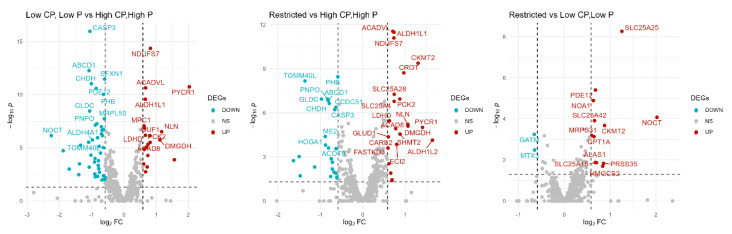
Volcano plots displaying the differential expression of 804 mitochondrial-associated genes for Low CP-P vs. High CP-P; Restricted vs. High CP-P; and Restricted vs. Low CP-P (n = 8 per group). Vertical dashed lines mark |log_2_FC| = 0.585 (1.5-fold); the horizontal dashed line marks and α of 0.05. Up to 30 of the most significant genes in each panel (lowest *p*-values) are labeled.

**Figure 2:**
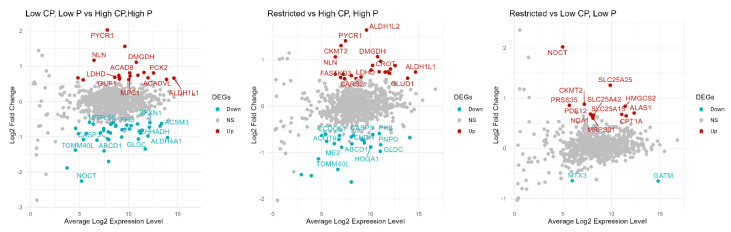
Mean-average (MA) plots of 804 mitochondrially associated genes for Low CP-P vs. High CP-P; Restricted vs. High CP-P; and Restricted vs. Low CP-P (n = 8 per group). Highlighted genes in red and blue are DEGs with |log_2_FC| = 0.585 (1.5-fold) and an α of 0.05. Up to 30 most significant (lowest *p*-value) genes per contrast are annotated.

Of the 1036 mitochondrial-associated genes cataloged in MitoCarta 3.0, we detected 804 in ovine liver. Eighty-three genes were differentially expressed (padj < 0.05; |log_2_ FC| ≥ 0.585) in at least one dietary contrast. Differential expression patterns are shown in volcano and mean-average (MA) plots (Figure 1 and Figure 2).

### 3.1. Hierarchical Clustering Analysis

**Figure 3:**
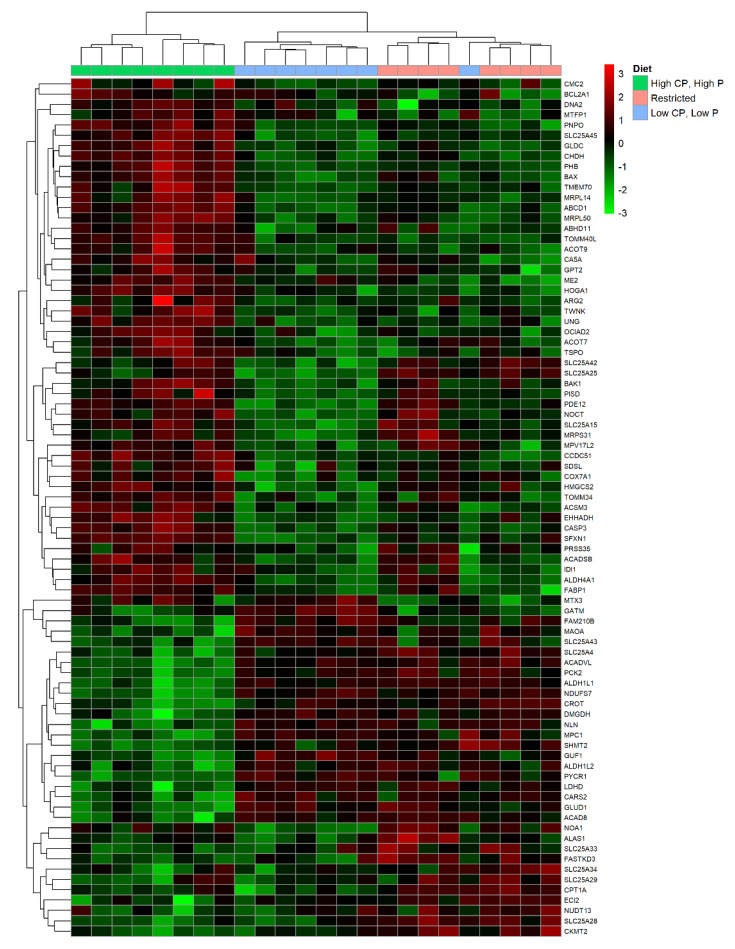
Hierarchical clustering heatmap of the 83 mitochondrially associated differentially expressed genes (DEGs) identified in at least one pairwise comparison between three dietary treatments. Each column represents an individual sheep liver sample (n = 24), colored by dietary group: High CP-P (green), Restricted (red), and Low CP-P (blue). Rows represent individual DEGs, with expression values z-score normalized across samples (red: high expression; green: low expression).

Hierarchical clustering of the 24 liver transcriptomes using the 83 mitochondrial DEGs showed clear grouping by diet (Figure 3). All High CP-P samples formed a distinct branch, while Restricted and Low CP-P samples resolved into separate subclusters that split away from the High CP-P group, consistent with the direction of the pairwise contrasts. No strong outliers were evident.

### 3.2. Network Overlays Highlight Diet-Specific Mitochondrial Modules

**Figure 4:**
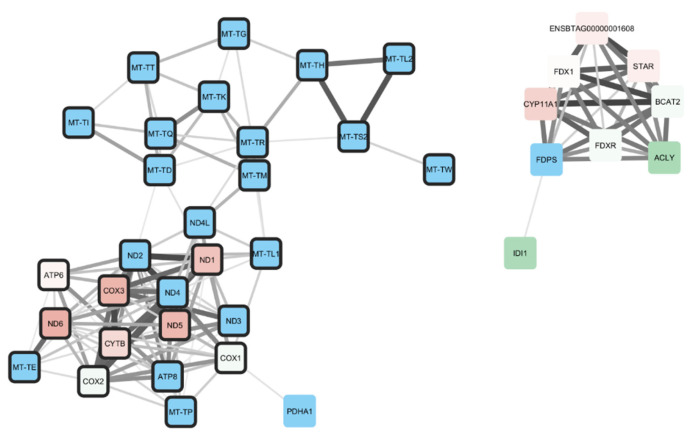
Transcriptomic changes (log_2_FC) from the Low CP-P vs. High CP-P contrast overlaid onto a previously developed bovine mitochondrial co-expression network [19]. The panel shows the mtDNA genome and biosynthesis subnetworks (nodes: mitochondrial proteins; edges: co-expression). Node color reflects log_2_FC (red = up, blue = not mapped, green = down) in CP/P deficiency.

**Figure 5:**
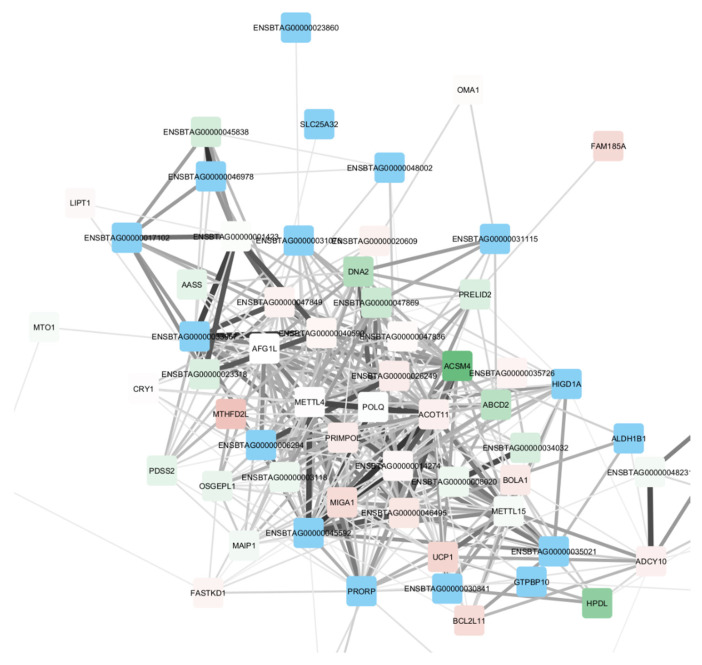
Transcriptome changes (log_2_FC) from the Low CP-P vs. High CP-P contrast overlaid onto a previously described bovine mitochondrial co-expression network [19]. The panel shows the mtDNA genome control and maintenance subnetwork (nodes: mitochondrial proteins; edges: co-expression). Node color reflects log_2_FC (red = up, blue = not mapped, green = down) in CP/P deficiency.

Overlaying log_2_FC from the Low CP-P vs. High CP-P contrast onto a bovine mitochondrial co-expression network identified some module level responses (Figure 4 and Figure 5). Network mapping confirmed a broad upregulation of the mtDNA-encoded OXPHOS/tRNA module, whereas the macromolecular biosynthesis subnetwork showed a mixed regulation across its components [19].

### 3.3. Pathway-Level Responses to Diet

***Low CP-P vs. High CP-P***

Thirty-eight DEGs mapped to eight enriched mitochondrial pathways. The most prominent enrichments occurred in amino-acid metabolism (16 DEGs of 72 genes, P = 2.8 × 10^−5^; mean log_2_ FC = −0.17). In particular, proline metabolism (2 of 4 genes, P = 0.03, mean log_2_ FC = +0.53), and folate/one-carbon metabolism (6 of 18 genes, P = 1.35 × 10^−3^; +0.41) were broadly upregulated in the Low CP-P group. Additional but smaller effects were evident in serine metabolism (3 of 4 genes, P = 1.58 × 10^−3^; −0.16), glycine metabolism (4 of 11 genes, P = 6.6 × 10^−3^; −0.06), vitamin metabolism (7 of 42 genes, P = 0.03; +0.23), and choline + betaine metabolism (2 of 5 genes, P = 4.9 × 10^−2^; +0.05). Collectively, these findings suggest coordinated downregulation of amino-acid catabolism and selective activation of one-carbon supply under protein-phosphorus deficiency.

***Restricted vs. High CP-P***

The energy-restricted group compared to those fed an adequate diet yielded ten enriched pathways from thirty-seven DEGs. Amino-acid metabolism showed moderate enrichment (12 of 72 genes, P = 4.4 × 10^−4^; +0.03) with no clear direction in regulation. The mitochondrial SLC25A transporter family was over-represented (6 of 40 genes, P = 2.4 × 10^−3^; +0.33), and TCA-associated enzymes exhibited a modest response (3 of 14 genes, P = 4.3 × 10^−2^; −0.22). Enriched pathways with the largest average expression differentiation include folate/one-carbon (5 of 18 genes, P = 2.6 10^−3^; +0.71) and vitamin metabolism pathways (6 of 42 genes, P = 0.03; +0.45), mirroring the pattern seen in protein-phosphorus deficiency.

***Restricted vs. Low CP-P***

Three pathways met the enrichment threshold from five detected DEGs. The SLC25A family remained prominent (3 of 40 genes, P = 2.8 × 10^−2^; +0.86). Nucleotide metabolism (3 of 29 genes, P = 1.2 × 10^−2^; +0.49) and creatine metabolism (2 of 2 genes, P = 2.8 × 10^−4^; +0.11) were unique to this comparison.

***Shared and unique responses***

**Figure 6:**
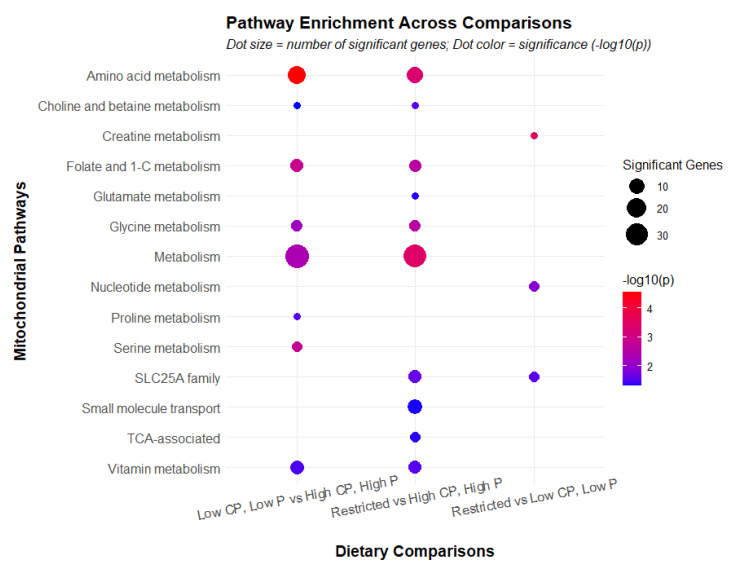
Dot plot of statistically significant mitochondrial metabolic pathways discovered through functional enrichment analysis within three contrasts between 24 merino wethers given a High CP-P diet, Low CP-P diet, and a feed-restricted (Restricted) diet (n = 8 per group).

No pathway was enriched in all three contrasts. Six pathways, including amino-acid metabolism, folate/one-carbon metabolism, glycine metabolism, choline and betaine metabolism, vitamin metabolism, and the broad metabolism parent set, were enriched in both Low CP-P and energy-restricted contrasts compared to the High CP-P diet fed sheep. The enrichment of genes related to the SLC25A transporter family was shared exclusively by the two contrasts to the energy-restricted diet group (Restricted v High CP-P; Restricted v Low CP-P). Proline and serine metabolism were unique to the Low CP-P v High CP-P contrast, whereas glutamate metabolism, small-molecule transport, and TCA-associated enzymes were unique to the Restricted v High CP-P contrast. A summary of enriched mitochondrial pathways across the three comparisons is shown in Figure 6.

### 3.4. Differentially Expressed Genes Within Enriched Pathways

**Figure 7:**
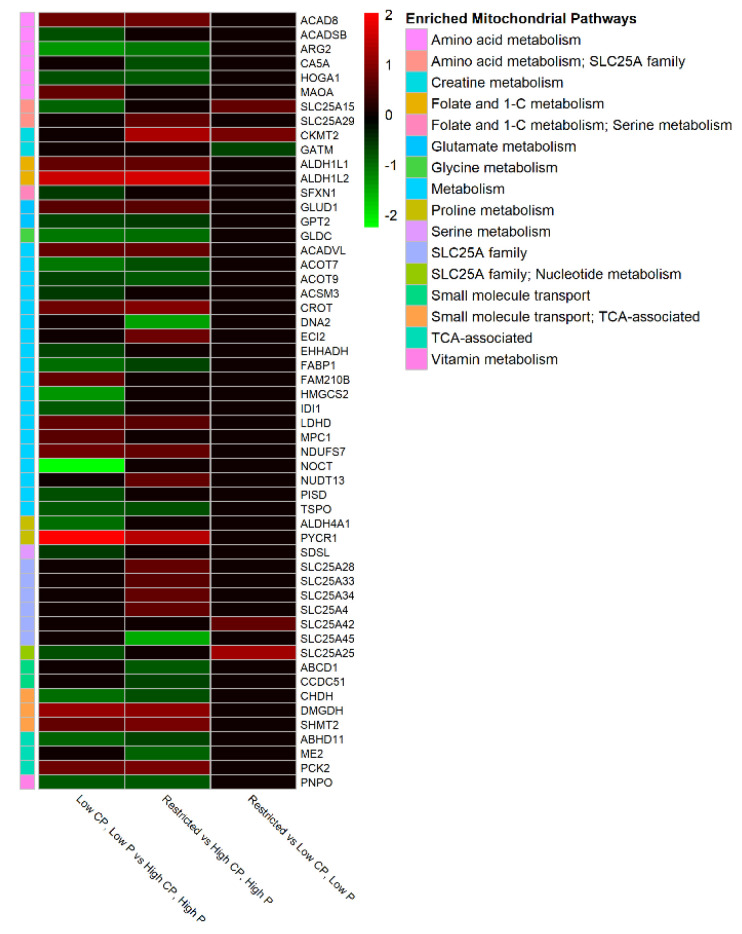
Heatmap of 54 mitochondrially associated DEGs found across three contrasts of three groups of wethers (n = 8 per group) fed either a High CP-P; Low CP-P, or a Restricted intake diet. DEGs are displayed with row annotations that indicate the two most specific MitoCarta enriched pathway assignments for that gene. Rows represent individual DEGs, with log_2_FC expression values across samples (red: high expression; green: low expression).

Fifty-four DEGs resided in at least one enriched pathway and form the functional core of the mitochondrial response in this study. The gene-level changes within these enriched pathways across the three comparisons are visualized in Figure 7.

***Low CP-P vs. High CP-P***

Sixteen DEGs fell within amino-acid metabolism, with ten repressed (mean log_2_ FC = −0.17). Key examples included *ACADSB* (−0.73), *ALDH4A1* (−0.98), *DMGDH* (+1.11), *PYCR1* (+2.03), and *SLC25A15* (−0.90). Folate/one-carbon DEGs that were broadly upregulated (mean +0.41); *ALDH1L2* (+1.56) and *SHMT2* (+0.74) typified this response. Two mitochondrial transporters, *SLC25A15* (ornithine transporter) and *SLC25A25* (adenine nucleotides)*,* were downregulated.

***Restricted vs. High CP-P***

Thirty-seven DEGs were identified. Serine metabolism was on average induced (+0.52), and folate/one-carbon genes were again elevated (+0.50). Transcripts for five SLC25A transporters (*SLC25A4*, *SLC25A28*, *SLC25A29*, *SLC25A33*, *SLC25A34*; transporting ADP/ATP, ions, amino acids, pyrimidines, and uncharacterized, respectively) were upregulated (mean +0.68), whereas *SLC25A45* (uncharacterized transporter) was suppressed (−1.50). Upregulation of *CKMT2* (+1.30) and repression of *DNA2* (−1.48) suggest shifts in creatine-related energy transfer and mtDNA maintenance during energy restriction.

***Restricted vs. Low CP-P***

Five genes were identified in the enriched pathways between the two nutrient-limited states. Three SLC25A carriers (*SLC25A15*, +0.68; *SLC25A25*, +1.26; *SLC25A42*, +0.66; transporting orinithine, adenine nucleotides, and CoA, respectively) were induced, with SLC25A15/25 reversing their repression under the Low CP-P vs. High CP-P comparison. The last two were creatine-pathway genes (*GATM*, −0.65; *CKMT2*, +0.88) and differentially regulated.

***Shared and contrast-specific genes***

Twenty-six genes were differentially expressed in at least two contrasts. Upregulation of *ALDH1L1* and *ALDH1L2*, *SHMT2*, and *PYCR1* related to one-carbon and proline pathways in the Low CP-P and Restricted group compared to the High CP-P group. Bidirectional regulation of *SLC25A15* and *SLC25A25* where they are repressed (−0.90, −0.79) in sheep experiencing CP and P deficiency but induced during CP and P adequate energy restriction (+0.68, +1.25) when compared to High CP-P group. There were no genes involved in the enriched pathways that were shared between all three contrasts.

***SLC25A carrier module***

Nine SLC25A transcripts were differentially expressed, split between two, six, and three transporters in the three contrasts (Low CP Low P v High CP High P, Restricted v High CP High P, Restricted v Low CP Low P), with mean log_2_ FC values of −0.84, +0.33, and +0.87, respectively. The gene encoding for *SLC25A45* lowered the earlier reported average mean log_2_ FC of this carrier module in the Restricted vs. High CP-P contrast from +0.68 to +0.33.

## 4. Discussion

In this study, Merino wethers showed both shared and distinct hepatic mitochondrial gene expression shifts in response to CP-P deficiency and matched ME intake feed restriction. Relative to sheep fed a High CP-P diet, both the Low CP-P and Restricted groups demonstrated similar mitochondrial transcriptomic responses (38 DEGs, 8 enriched pathways; 37 DEGs, 10 enriched pathways; respectively), whereas few differences were observed between the Restricted and Low CP-P groups (five DEGs, three enriched pathways). This pattern suggests that reduced metabolizable energy intake was the dominant driver of the hepatic mitochondrial response. Across both low-intake treatments, the clearest shared signal was upregulation of folate-mediated one-carbon metabolism, whereas contrast-specific differences were most evident in mitochondrial transporter-related genes, particularly within the SLC25A family. Together, these findings indicate that low ME intake accounted for much of the mitochondrial transcriptional response, while CP-P deficiency contributed to a smaller, more specific component of variation.

### 4.1. One-Carbon Metabolism Is a Shared Adaptive Hub of Energy Restriction

Folate-mediated one-carbon (1C) metabolism emerged as a consistently upregulated pathway in both CP and P deficient and energy-restricted sheep compared to the group fed the High CP-P diet. Both the Low CP-P sheep and Restricted sheep exhibited upregulation in the folate/1C pathway (mean log_2_FC = +0.41 and +0.50, respectively). This response occurred within a broader set of shared pathway enrichments, including amino-acid, glycine/serine, choline-betaine, and vitamin metabolism, reinforcing the view that reduced ME intake imposed a shared mitochondrial transcriptional response.

One-carbon metabolism plays an essential role in various biosynthetic reactions, including nucleotide synthesis, methionine regeneration, and provision of reducing equivalents (NADPH) for antioxidant defense [21,22,23]. The core metabolite, 5,10-methylene tetrahydrofolate (5,10-methylene-THF), serves as a critical junction molecule, linking serine and glycine metabolism to dTMP, purine, and methionine synthesis.

Several key enzymes regulating the 1-C pathway exhibited significant differential expressions under low ME intake. Notably, *SHMT2*, which encodes mitochondrial serine hydroxymethyltransferase, was markedly upregulated, suggesting enhanced conversion between serine and THF with glycine and 5,10-methylene-THF. Simultaneously, *GLDC*, which translates to a glycine cleavage enzyme responsible for glycine degradation, was downregulated. This combination of *SHMT2* and *GLDC* expression suggests that glycine conservation is favored, reinforcing the supply of glycine, which is considered a conditionally essential amino acid in low CP and feed-restricted diets, as it plays a broad role in the synthesis of proteins, purines, porphyrins, creatine, and glutathione (GSH) [24,25,26]. The significant upregulation of *DMGDH*, which catalyzes dimethylglycine conversion toward sarcosine, and ultimately glycine, further complements this glycine conservation strategy.

**Figure 8:**
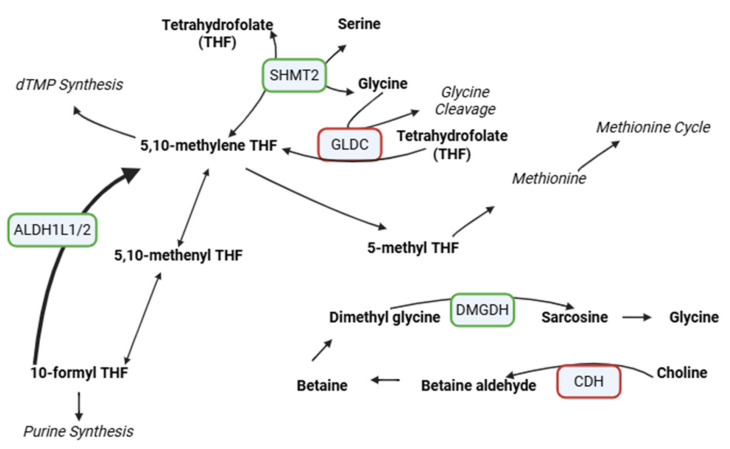
Folate/one-carbon (1-C) pathway genes differentially expressed in liver in the Low CP-P vs. High CP-P comparison (n = 8/group). Enzymes with significant differential expression (padj ≤ 0.05; |log_2_FC| ≥ 0.585) are boxed and color-coded: upregulated (green), SHMT2, ALDH1L1/ALDH1L2, DMGDH; downregulated (red), GLDC, CHDH (choline dehydrogenase). Arrows indicate major THF-linked fluxes connecting serine/glycine metabolism to dTMP and methionine synthesis.

Moreover, *ALDH1L1* and *ALDH1L2*, pivotal in converting 10-formyl-THF back into THF while producing NADPH, was significantly elevated. This transcriptional shift suggests prioritizing THF recycling to maintain the 1-C pool, while also sustaining mitochondrial NADPH, which diverts 10-formyl-THF from purine synthesis, and possibly bolstering antioxidant defenses under oxidative stress conditions associated with nutrient deficiencies [27,28]. Concurrently, *CHDH* downregulation indicates reduced choline catabolism, which may preserve phosphatidylcholine integrity in cell membranes, crucial during phosphorus deficiency [29]. A diagram visualizing how the one-carbon metabolism pathway is impacted in both the Low CP-P vs. High CP-P and Restricted vs. High CP-P comparisons is shown in Figure 8.

### 4.2. Arginine and Proline Pathway Response to Low Metabolizable Energy Intake

The arginine and proline metabolism pathway suggested a conservatively oriented response under low ME intake (voluntarily via a low CP-P diet, and a restricted nutritionally adequate diet). In both contrasts to High CP-P, lower *ARG2* expression indicates reduced conversion of arginine to ornithine and urea, suggesting lower nitrogen loss or flux. At the same time, upregulation of *PYCR1* and simultaneous downregulation of *ALDH4A1* (only in Low CP-P vs. High CP-P) and *HOGA1* suggest greater retention of proline and hydroxyproline through reduced catabolism and maintained synthesis. As proline and hydroxyproline make up about 23%, in addition to glycine comprising 33% of the AA content in collagen [30], these data may point to a collagen-preserving strategy. A previous study observed that feed-restricted lambs increased collagen content and gene expression of prolyl 4-hydroxylase α subunit (P4HA1) in semitendinosus muscle, which catalyzes hydroxyproline synthesis [31].

### 4.3. Energy Restriction and Low CP-P Induce Distinct Mitochondrial Transporters

A defining signature of energy restriction from this study was the induction of mitochondrial solute carrier (SLC25) genes, but the effect depended on the comparison. In Restricted vs. High CP-P, the SLC25A family was enriched (6 of 40 genes; mean log_2_FC = +0.33), and of those six, five DEGs for SLC25A transporters, *SLC25A4* (ANT1), *SLC25A28* (MFRN2), *SLC25A29* (basic/cationic amino-acid carrier, e.g., Arg/Lys/Orn), *SLC25A33* (PNC1), and *SLC25A34*, were co-induced (DEG-set mean log_2_FC = +0.68). By contrast, *SLC25A45* (orphan carrier) was the only repressed SLC25A gene in this contrast (−1.50 log_2_FC). *SLC25A34*, while mainly uncharacterized, mouse studies have linked this carrier to limited lipogenesis via beta-oxidation, and reduced mitochondrial mass and energy output [32]. ANT1 (ATP/ADP transporter) upregulation would accelerate ADP import and ATP export, supporting ATP turnover when substrates are scarce [33,34]. MFRN2 regulates iron ion transport for heme and iron-sulfur cluster synthesis and PNC1 is a carrier for pyrimidine nucleotides [35].

In Restricted vs. Low CP-P, a smaller transporter set, *SLC25A15* (ORC1), *SLC25A25* (SCaMC-2), *SLC25A42* (CoA transporter), was upregulated (+0.68, +1.26, +0.66 log_2_FC, respectively; mean = +0.86). It is also noteworthy that SLC25A15/25 specifically reversed their repression seen in Low CP-P vs. High CP-P (−0.90 and −0.79 log_2_FC, respectively). This suggests that sheep consuming a low CP-P diet may undergo a coordinated downregulation of these two mitochondrial carriers that are specific to CP and P deficiency. SLC25A25 is known as an ATP-Mg/Pi carrier, assisting in ATP efflux and synthesis. SLC25A15 is an ornithine carrier whose downregulation would be consistent with reduced urea-cycle flux. Whether this downregulation is representative of a strategy to conserve nitrogen or simply because of lower protein catabolism remains unclear. The expression of *SLC25A15* was unimpacted in the Restricted vs. High CP-P comparison, which suggests that while the Restricted group was consuming less ME, the urea cycle was not as impacted when compared to an adequate ad libitum diet. The observed lower voluntary intake, weight gain, plasma urea-N, and ruminal NH_3_-N under CP and P deficiency in ruminants [2,36,37] is consistent with, and would further reinforce, reduced hepatic amino-acid flux. Together, the patterns within this enriched pathway suggest that feed restriction of a nutritionally adequate diet elicited a transporter-centered mitochondrial response, whereas CP-P deficiency was associated with more selective transporter changes linked to nitrogen handling.

### 4.4. Symmorphosis and Metabolic Scope

The observed mitochondrial transcriptional adjustments align with the concept of symmorphosis, where metabolic capacity matches functional demand across species [38,39,40,41]. The reduced mitochondrial DNA copy numbers from these sheep suggests that mitochondrial mass, and thus, overall oxidative capacity, was lowered and is consistent to lower ADG [2,15]. Moreover, the transcriptomic data from this study points to lower ME intake, regardless of if due to CP-P deficiency or feed restriction, being associated with upregulated one-carbon metabolism pathways, suggesting a prioritization of methyl donor and NADPH-related metabolism in response to energetic stress. In contrast, feed-restricted sheep consuming an adequate CP-P diet experienced an upregulation of genes for several SLC25A mitochondrial transporters, which may be a compensatory response to energetic stress by prioritizing the shuttling of molecules and ions through mitochondria. Also, CP-P deficiency-induced energy restriction involved a selective downregulation of mitochondrial transporters linked to urea production and ATP shuttling. Together, these findings support the view that hepatic mitochondrial responses to nutrient limitations were selective and align with adjusting metabolism due to substrate supply and physiological demand.

### 4.5. Implications for Grazing Systems

Mitochondrial transcriptomic signatures identified here may provide useful candidate indicators of specific nutrient deficiencies. In particular, regulation of the mitochondrial transport gene *SLC25A15* was associated with CP-P deficiency and may help distinguish nutrient deficiency from reduced intake alone. Likewise, the shared induction of one-carbon metabolism under both low-intake treatments may indicate increased pressure to conserve glycine and sustain methyl-donor and NADPH supply, highlighting glycine or related one-carbon nutrients as candidate targets for future nutritional interventions in ruminants grazing nutrient-limited pastures. More broadly, these findings improve mechanistic understanding of how chronic CP and P deficiency, together with reduced intake, may constrain productivity in extensive grazing systems. These findings may provide a foundation for future targeted nutritional interventions and selection strategies for improving extensive livestock systems.

## Data Availability

The raw data generated in this experiment is available upon reasonable request at the discretion of the authors.

## Appendix A

**Table A1:** Ingredient and chemical composition of pellet diets fed to forty growing wethers (n = 8/group) during the feeding trial. Adapted from Innes et al. (2023) [2].

Item	High CP, High P Pellet	High CP, Low P Pellet	Low CP, High P Pellet	Low CP, Low P Pellet
*Ingredient Composition (% as fed)*				
Barley straw	55.0	55.6	57.0	57.5
Sugar	23.0	23.0	25.0	25.0
Cassava starch	10.0	10.0	13.0	13.2
Gluten	9.0	9.0	2.5	2.5
BioFos ^1^	0.9	0.0	1.0	0.1
Fine limestone (38% Ca)	0.2	0.5	0.1	0.4
Urea	0.7	0.7	0.0	0.0
Sodium bicarbonate	0.5	0.5	0.5	0.5
Gypsum	0.3	0.3	0.4	0.4
Potassium chloride	0.3	0.3	0.3	0.3
Magnesium oxide	0.2	0.2	0.2	0.2
Trace mineral mix	0.1	0.1	0.1	0.1
*Chemical Composition (g/kg DM)*				
OM	922	929	921	919
CP	114	115	56	52
P	2.2	0.7	2.5	0.7
Ash-free NDF	332	372	364	369

^1^ BioFos is a monocalcium phosphate supplement and is a registered trademark of The Mosaic Company, Florida, USA; sourced from Ridley AgriProducts Pty Ltd., Pakenham, VIC, Australia.
